# Sleep-related breathing disorders and gait variability: a cross-sectional preliminary study

**DOI:** 10.1186/1471-2466-14-140

**Published:** 2014-08-23

**Authors:** Sébastien Celle, Cédric Annweiler, Richard Camicioli, Jean-Claude Barthélémy, Frédéric Roche, Olivier Beauchet

**Affiliations:** 1 Service de Physiologie Clinique et de l’Exercice, CHU Saint-Etienne; EA 4607 “SNA EPIS” Faculté de Médecine Jacque Lisfranc UJM, PRES Université de Lyon, Lyon 42023, France; 2 Department of Neuroscience, Division of Geriatric Medicine, UPRES EA 4638, UNAM, Angers University Hospital, 49933 Angers, Cedex 9, France; 3 Center for Functional and Metabolic Mapping, Robarts Research Institute, Department of Medical Biophysics, Schulich School of Medicine and Dentistry, the University of Western Ontario, London, Ontario, Canada; 4 Department of Medicine, Glenrose Rehabilitation Hospital, University of Alberta, Edmonton, Alberta, Canada

**Keywords:** Gait disorders, Sleep-related breathing disorders, Older adults

## Abstract

**Background:**

Sleep-related breathing disorders (SRBDs) provoke cognitive and structural brain disorders. Because these disorders have been associated with unsafe gait characterized by an increase in stride-to-stride variability of stride time (STV), we hypothesised that SRBDs could be associated with an increased STV. The aim of this study was to examine the association between SRBDs and STV in French healthy older community-dwellers.

**Methods:**

A total of 49 participants (mean age 69.6 ± 0.8years; 65.2% female) were included in this cross-sectional study. All participants, who were free of clinically diagnosed SRBDs before their inclusion, had a nocturnal unattended home-sleep assessment. There were separated in three group based on apnea + hypopnea index (AHI): AHI <15 defining the absence of SRBD, AHI between 15–30 defining mild SRBD, and AHI >30 defining moderate-to-severe SRBD. Coefficient of variation of stride time, which is a measure of STV, was recorded while usual walking using SMTEC® footswitches system. Digit span score was used as a measure of executive performance. Age, gender, body mass index (BMI), number of drugs daily taken, vision, proprioception, history of falls, depression symptoms, global cognitive functioning were also recorded.

**Results:**

STV and BMI were higher in participants with mild SRBDs (P = 0.031 and P = 0.020) and moderate-to-severe SRBDs (P = 0.004 and P = 0.002) compared to non-SRBDs. STV positively correlated with AHI (P = 0.036). Lower (i.e., better) STV was associated with the absence of SRBDs (P = 0.021), while greater (i.e., worse) STV was associated with moderate-to-severe SRBD (P < 0.045) but not with mild SRBD (P > 0.06).

**Conclusion:**

Our results show a positive association between STV and SRBDs, with moderate-to-severe SRBD being associated with greater gait variability. This association opens new perspectives for understanding gait disorders in older adults with SRBDs and opens the door to treatments options since SRBDs are potential treatable factors.

## Background

Sleep-related breathing disorders (SRBDs) refer to a large spectrum of abnormal respiratory patterns ranging from habitual snoring to obstructive sleep apnea (OSA) and central sleep apnea, occurring while sleeping and resulting in an abnormal reduction in gas exchange (i.e., hypoxemia) [1,2]. The prevalence of SRBDs is high and increases with age and obesity [1-3]. The most prevalent is OSA [2,4]. For instance in the United-States of America, it has been estimated that OSA affects approximately 25% of adults aged 65 years and over [2]. In addition, the prevalence of non-diagnosed SRBDs in older community-dwellers is high and may reach 54%, as recently shown by Sforza et al. [4].

SRBDs are important causes of morbidities such as cognitive impairment, headaches, hypertension, and daytime fatigue mediated in part by chronic-intermittent hypoxia [1-4]. Cognitive, metabolic and structural brain disorders resulting from chronic-intermittent hypoxia have been previously reported in animals and humans [4,5]. The consequence of SRBD chronic-intermittent hypoxia on gait changes in older adults has been few examined, whilst there is a strong rational for such an association. Indeed, it is well-recognized that gait changes are strongly related to impaired cognition and brain lesions [6,7]. Recently, Allali et al. [8] reported that gait abnormalities in patients with OSA were partially managed with continuous positive airway pressure (CPAP), as illustrated by an improvement of gait performance after CPAP. It was suggested that this result could be explained by CPAP-related brain changes leading to a safer gait. Unfortunately, in this study, gait variability, and more specifically stride-to-stride variability of stride time (STV) was not examined, although it has been identified as a dependable biomarker of the rhythmic stepping mechanism related to cognitive performance and brain structures integrity [6,7]. Greater STV reflects unsafe gait and exposes to several adverse consequences such as falls, fractures and disability in older adults [6].

Because SRBDs may induce cognitive, metabolic and structural brain disorders, and because these disorders have been associated with unsafe gait, specifically an increase in STV, we hypothesised that SRBDs could be associated with an increased STV. Exploring this association may be helpful to better understand gait disorders in older adults with SRBDs. The aim of this study was to examine the association between SRBDs and STV in French healthy older community-dwellers free of clinically diagnosed SRBDs.

## Methods

### Participants

A total of 49 participants (mean age ± standard deviation, 69.6 ± 0.8 years [range 67–73]; 65.2% female) from the PROgnostic indicator OF cardiovascular and cerebrovascular events (PROOF) cohort study [4] were included in this study after having given their written informed consent for research. The entire study protocol was approved by the local Ethical Committee of Saint-Etienne, France. Inclusion criteria for the present analysis were: age ≥ 65 years, no chronic condition influencing gait variability, no clinically diagnosed SRBD and no cognitive decline. We specifically focused on a sample of participants with no clinically diagnosed SRBD in order to examine the consequence of SRBDs-related chronic-intermittent hypoxia on gait changes without any treatment effect [8]. In addition, we excluded participants with neurological disease including Parkinson’s disease, cerebellar disease, myelopathy, peripheral neuropathy and cognitive impairment (i.e., mild cognitive impairment and dementia); severe depression symptoms (score of self-rated Pichot depression scale [QD2A questionnaire] >6) [9]; major orthopaedic diagnoses involving the lumber vertebra, pelvis or lower extremities; use of walking aids. Finally, two more participants were excluded because they had aberrant values of AHI and STV.

### Sleep-related breathing disorders assessment

All participants had a nocturnal unattended home-sleep assessment using a polygraphic system (HypnoPTT, Typco Healthcare, Purain Bennett). A recording was considered acceptable if ≥5 h of recording without missing data on respiratory signals or SpO2 was obtained. Hypopnea was defined as ≥50% reduction in airflow from baseline value lasting ≥10s and associated with ≥3% oxygen desaturation. Apneas were defined as the absence of airflow on the nasal cannula lasting >10 s. The apnea + hypopnea index (AHI) was established as the ratio of the number of apneas and hypopneas per hour of recording. AHI <15 defined the absence of SRBDs, AHI between 15–30 defined mild SRBDs, and AHI >30 defined moderate-to-severe SRBDs [3].

### Gait assessment

STV was measured at steady-state self-selected walking speed in a 20-meter long corridor using the SMTEC® footswitches system (SMTEC®, Sport & Medical Technologies SA, Nyon Switzerland). To assure that gait parameters were collected while steady state walking, participants started walking at least 2 meters before reaching the 10-meter walkway and completed their walk at least two meters beyond it [10]. Participants walked one trial in a quiet, well-lit corridor wearing their own footwear. Coefficient of variation (CoV) (CoV = (standard deviation/ mean) × 100) of stride time (i.e., gait cycle duration defined as the time elapsed between the first contact of two consecutive footsteps of the same foot) was used as a measure of STV. The number of strides ranged from 12 to 24 steps by participants. Little is known about the test-retest reliability of STV but it has been recently reported that the immediate reliability of the CoV of stride time while single- and dual-tasking is slight to poor but better in cognitively healthy individuals than in those with cognitive decline [11]. STV was used either as a continuous variable or as a categorical variable based on tertilization: lowest tertile (STV < 1.6%; 1.2 ± 0.3% [range 0.6-1.5]; n = 16), intermediate tertile (1.6-2.0%; 1.7 ± 0.1% [range 1.6-1.9]; n = 16) and highest tertile (>2.0%; 2.7 ± 0.7% [range 2.0-5.9]; n = 17).

### Clinical assessment

Participants underwent a full medical examination at a couple of days before SRBDs and gait assessment. Age, gender, height (m), weight (kg) and the number of drugs daily taken were recorded. Body mass index (BMI, in kg/m^2^) was calculated. Overweight was defined as ≥ 25 kg/m^2^. Lower-limb proprioception was evaluated with a 64 Hz graduated tuning fork placed on the tibial tuberosity, and graded from 0 (i.e., worst performance) to 8 (i.e., best performance). The mean value obtained for the left and right sides was used in the present data analysis. Lower-limb osteoarthritis was also recorded. Distance binocular vision was measured at 5 m with a standard Monoyer letter chart [12]. Vision was assessed with corrective lenses on if regularly used by the participant. The participants were interviewed using a standardized questionnaire, gathering information on the history of falls over the past year [13]. Depressive symptoms were assessed using the QD2A questionnaire [9]. Cognition was evaluated using common neuropsychological tests. First, global cognitive efficiency was evaluated with Folstein’s Mini-Mental State Examination (MMSE) score [14]. Second, verbal episodic memory was evaluated using the French version of the Free and Cued Selective Reminding Test (FCSRT) [15]. We used as outcome value the total delayed recall and free delayed recall, higher value denoting better memory. Digit span score examine the ability to recall a sequence of numbers forward and backward in corrected order immediately after its presentation. This management of information is based on monitoring and updating processes which has been recently related to STV [16].

### Statistics

The participants’ characteristics were summarized using means and standard deviations or frequencies and percentages, as appropriate. For the present analysis, participants were separated into 3 groups based on the AHI score: <15 (i.e., non-SRBDs), 15–30 (i.e., mild SRBDs), and >30 (i.e., moderate-to-severe SRBDs). Comparisons between groups were performed using Kruskal-Wallis, Mann–Whitney or Chi-square test, as appropriate. Spearman’s rho test was used to examine the correlation between STV and AHI score. Multiple logistic regression analyses was performed to specify the association between the highest tertile (i.e., worst) of STV and the three levels of AHI score (independent variables) adjusted for BMI dichotomized (i.e., >25 kg/m^2^) and digit span score. P-values <0.05 were considered as statistically significant. All statistics were performed using SPSS (version 19.0; SPSS, Inc., Chicago, IL).

## Results

As shown in Table 1, CoV of stride time and BMI were higher in participants with mild SRBDs (P = 0.031 and P = 0.020) and moderate-to-severe SRBDs (P = 0.004 and P = 0.002) compared to non-SRBDs. There was no significant difference for the other clinical characteristics.

**Table 1:** Clinical characteristics of participants according to their apnea-hypopnea index (n = 49)

	Apnea-hypopnea index*			**P-Value**			
	**< 15 (n = 23)**	**15-30 (n = 19)**	**>30 (n = 7)**	**Overall**^**†**^	**<15 versus 15-30**^**‡**^	**<15 versus >30**^**‡**^	**15-30 versus >30**^**‡**^
Age, mean ± SD (years)	69.6 ± 0.8	69.8 ± 0.7	70.6 ± 1.4	0.174	-	**-**	-
Female, n (%)	15 (65.2)	8 (42.1)	4 (57.1)	0.284	-	-	-
Body mass index (kg/m^2^),							
Mean ± SD	24.1 ± 2.5	26.6 ± 3.2	28.4 ± 2.44	**0.003**	**0.020**	**0.002**	0.231
>25, n (%)	10 (43.5)	13 (68.4)	7 (100.0)	**0.021**	0.314	**0.013**	0.314
Number of drugs taken daily, mean ± SD	1.9 ± 1.8	2.2 ± 1.52.3	2.3 ± 1.1	0.710	-	-	-
Vision^¶^ (/10), mean ± SD	8.3 ± 2.5	8.3 ± 3.3	8.0 ± 2.0	0.634	**-**	**-**	-
Lower-limb proprioception^#^ (/8), mean ± SD	5.0 ± 1.7	4.7 ± 1.3	4.0 ± 1.4	0.219	**-**	**-**	-
Lower-limb osteoarthritis, n (%)	1 (4.3)	2 (10.5)	1 (14.3)	0.701	**-**	**-**	-
History of falls in the past year, n (%)	7 (30.4)	5 (26.3)	1 (14.3)	0.703	**-**	**-**	-
Depression^**^ (/13), mean ± SD	1.8 ± 2.1	0.9 ± 1.1	2.0 ± 1.8	0.361	**-**	**-**	-
Mini mental status examination ††score (/30), mean ± SD	29.1 ± 1.1	29.3 ± 0.7	28.9 ± 0.9	0.509	**-**	**-**	-
Total delayed recall and free delayed recall ‡‡ score (/48), mean ± SD	47.0 ± 2.0	47.6 ± 0.7	47.1 ± 1.5	0.616	**-**	**-**	-
Digit span score^¶¶^, mean ± SD	10.5 ± 1.9	10.6 ± 1.9	9.1 ± 0.9	0.106	-	-	-
Coefficient of variation of stride time (%), mean ± SD	1.5 ± 0.5	2.2 ± 0.9	2.3 ± 0.5	**0.010**	**0.031**	**0.004**	0.395

*<15 = no sleep-related breathing disorder (SRBD), 15-30 = mild SRBD, and >30 = moderate-to-severe SRBD.

^†^Comparison based on Kruskal-Wallis or Chi-square test, as appropriate.

^‡^Comparison based on Mann–Whitney test.

^¶^Binocular vision acuity at distance of 5 m with a Snellen letter test chart.

^#^Mean value of left and right sides, based on graduated tuning fork placed on the tibial tuberosity.

**Depressive symptoms measured using the QD2A questionnaire including 13 questions with scores ranging from 0 to 13 points.

††Cognitive test exploring global cognitive function with score at 30 corresponding to best performance (i.e., healthy cognitive status).

‡‡Grober & Buschke test exploring episodic memory with score at 48 corresponding to best performance (i.e., no episodic memory disorders).

^¶¶^Total number of digits that a participant can absorb and recall in correct forward and backward serial orders after hearing them.

P significant (<0.05) indicated in bold.

STV positively correlated with AHI (P = 0.036). When AHI and STV were considered as categorical variables, the logistic regression models showed that the highest tertile of STV was inversely associated with the absence of SRBDs defined as being in the group of participants with AHI score <15 (unadjusted OR = 0.21 with 95% confident interval (CI) = [0.05; 0.74] and P = 0.021 when using participants with mild SRBDs and those with moderate-to-severe SRBDs as the referent group) and positively associated with moderate-to-severe SRBDs (unadjusted OR = 6.25 with 95% CI = [1.17; 48.04] and P = 0.042 when using participants without SRBDs and those with mild SRBDs as referent group) (Table 2). After adjustment for covariables (i.e., overweight [BMI ≥ 25 kg/m^2^] and digit span score), moderate-to-severe SRBDs (adjusted OR = 16.98 with 95% CI = [1.74; 268.42] and P = 0.024) but not mild SRBDs (adjusted OR = 4.98 with 95% CI = [0.98; 32.31] P = 0.065) was positively associated with the highest tertile of STV. In addition, overweight (i.e., BMI ≥25 kg/m^2^) was associated with lower STV (adjusted OR = 0.13 with 95% CI = [0.01; 0.76] P = 0.039).

**Table 2:** Multivariate logistic regressions showing the association between the highest tertile (i.e., worst performance) of coefficient of variation of stride time (dependent variable) and the apnea-hypopnea index categorized into 3 levels of severity of sleep-related breathing disorders (ie, no disorder; mild disorder; moderate-to-severe disorder; independent variable) (n = 49)

	**Highest tertile of stride time variability**					
	**Model 1**^**†**^			**Model 2**^**‡**^		
	**OR**	**95% CI**	**P-value**	**OR**	**95% CI**	**P-value**
Apnea-hypopnea index:						
< 15	0.21	[0.05;0.74]	**0.021**	1.00 (Ref*)		
15-30	1.70	[0.51;5.72]	0.388	4.98	[0.98;32.31]	0.065
>30	6.25	[1.17;48.04]	**0.042**	16.98	[1.74;268.42]	**0.024**
BMI ≥ 25 kg/m^2^	0.86	[0.26;2.92]	0.802	0.13	[0.01;0.76]	**0.039**
Digit span score	0.72	[0.48;1.02]	0.077	0.71	[0.45;1.06]	0.108

CI = confidence interval; BMI = body mass index; OR: odds ratio; *Apnea-hypopnea index <15 used as reference level; ^†^Separated model for Apnea-hypopnea index, body mass index and digit span score; ^‡^Full adjusted model.

P significant (<0.05) indicated in bold.

## Discussion

Our results show that a positive association between STV and SRBDs, moderate-to-severe SRBDs being associated with greater (i.e., worse) gait variability.

To the best of our knowledge this study is the first to identify a significant association between CoV of stride time and SRBDs severity in older community-dwellers. This result is in concordance with a recent study that underscored that patients with OSA had gait abnormalities and that these gait abnormalities could be treated by CPAP [8]. In this study, mean values of spatio-temporal gait parameters were measured before and after 8 weeks of CPAP. It was observed a significant improvement of step time and stance time before CPAP. SRBDs are a cause hypertension provoked by chronic-intermittent hypoxia [1-4], which may lead to cerebrovascular brain lesions. High blood pressure level is well-known to provoke ischemic and/or haemorrhagic lesions in white and gray matters but also brain volume reduction [17]. Ischemic as well neurodegenerative lesions of the brain have been reported with chronic episodic hypoxia during sleep in animals and humans [5]. STV is a measure of the reliability of lower-limb movements which depends on higher-levels of gait control involving brain structures that may be affected by SRBDs-related brain lesions [6,7].

As previously reported, we also found that the severity of SRBDs was positively associated with increased BMI [18]. But we reported that overweight was associated with lower STV, which seems in discordance with previous results. Indeed, there is a growing body of evidence that excessive body weight is inseparably connected to postural instability. It has been reported that obese individuals exhibited poorer performance on both static and dynamic posture tasks with greater postural motion in the medial/lateral direction when compared to non-obese individuals [19]. BMI has also been used to predict postural stability [20]. Obese individuals show an inverse relationship between body mass/percentage of fat/total fat mass and clinical balance score. One possible explanation of our result is that previous studies underscored an association only with overweight-related dysfunction of postural control but not with impairment in control of the rhythmic stepping mechanism [6,7].

Our results did not confirm the hypothesis that SRBDs-related increase in STV is due to SRBDs-related dysexecutive function. The main explanation is related to the fact that all participants were cognitively healthy individuals with no significant between-group difference in terms of digit span score. Another explanation could be that increase in STV is not a consequence of dysexecutive function but rather a primary symptom of SRBDs. It has been previously reported that motor disorders, and more specifically gait disorders, may be the first symptom of neurodegenerative brain disease such as Alzheimer disease [21].

Our study has some limitations. First, the study was restricted to healthy volunteers who are likely not representative of community-dwelling older adults, probably more motivated with greater interests in personal health issues. Secondly, the cross-sectional design did not allow any causal inferences. Hence, longitudinal studies are needed to follow up on our initial promising results to address issues of causality. Thirdly, although we controlled for many characteristics likely to modify the association between STV and SRBDs, residual confounders may still be present. Fourthly, limitations of this study include a relative small number of participants. For instance, there were only 7 individuals with moderate-to-severe SRBDs (i.e., AHI >30), which could increase the risk of misinterpretation of the association with highest AHI scores. Unfortunately no power calculation was performed before the study.

## Conclusions

Moderate-to-severe SRBDs was associated with greater (i.e., worse) gait variability. Our findings provide a new insight into SRBDs’ adverse consequences. Reported SRBDs-related increase in gait variability opens new perspectives for understanding gait disorders in older adults with SRBDs and opens the door to treatment options since SRBDs are potential treatable factors. Future investigations are required to confirm these preliminary results and to investigate the underlying mechanisms and the causality of the association between SRBDs and gait disorders.

## Abbreviations

SRBDs: Sleep-related breathing disorders; OSA: Obstructive sleep apnea; CSA: Central sleep apnea; US: United-States; STV: Stride-to-stride variability of stride time; AHI: Apnea + hypopnea index; CoV: Coefficient of variation; BMI: Body mass index; CI: Confidence interval.

## Competing interests

The authors report no competing interest.

This study was supported by a grant from the French Minister of Health (Cellule Projet Hospitalier de Recherche Clinique National, Direction interrégionale de la Recherche Clinique, CHU Saint-Etienne; Appel d’Offre 1998 and Appel d’Offre 2002), by Associations SYNAPSE and ONDAINE.

## Authors’ contributions

OB has full access to all of the data in the study, takes responsibility for the data, the analyses and interpretation and has the right to publish any and all data, separate and apart from the attitudes of the sponsor. All authors meet all of the following criteria: (1) contributing to the conception and design, or analyzing and interpreting data; (2) drafting the article or revising it critically for important intellectual content; and (3) approving the final version to be published.

## Pre-publication history

The pre-publication history for this paper can be accessed here:

http://www.biomedcentral.com/1471-2466/14/140/prepub
